# Cultivated Land Change, Driving Forces and Its Impact on Landscape Pattern Changes in the Dongting Lake Basin

**DOI:** 10.3390/ijerph17217988

**Published:** 2020-10-30

**Authors:** Junhan Li, Kaichun Zhou, Huimin Dong, Binggeng Xie

**Affiliations:** 1 College of Resources and Environmental Sciences, Hunan Normal University, Changsha 410081, Hunan, China; junhanl@163.com (J.L.); zhoukaichun@hunnu.edu.cn (K.Z.); 2 College of Resources and Environment, Shandong Agricultural University, Taian 271018, China; donghui_min@163.com

**Keywords:** cultivated land changes, driving force, landscape pattern, trend analysis

## Abstract

Comprehending the dynamic change characteristics of land use/cover and the driving factors causing the change are prerequisites for protecting land resources. This paper analyzes changes in cultivated land, the driving factors that cause them, and their tremendous impact on landscape pattern changes in the Dongting Lake Basin. For this purpose, we used mathematical statistics, buffer analysis, trend analysis, landscape pattern index, and logistic regression model to analyze the land use data of the study area from 1980 to 2018. The results show that the cultivated land showed a decreasing trend, with the total area decreased by 4.76% (or 716.13 km^2^) from 1980 to 2018, and the activity of mutual transformation with other land use types decreased. The spatial distribution pattern of cultivated land and landscape shows the change characteristics gradually from Dongting Lake to the surroundings. Among the driving factors of cultivated land changes, the influence of human activities was gradually increasing, while the natural factors were decreasing. The cultivated land landscape pattern index and the overall landscape pattern index have a significant positive correlation, showing relatively consistent change trend and spatial distribution characteristics. We believe that the decrease of cultivated land area has a certain relationship with the increase of landscape fragmentation in the Dongting Lake Basin. Our research is expected to provide a reference for strengthening regional cultivated land management and rational development and utilization of regional land resources.

## 1. Introduction

Land use and land cover change (LUCC) is affected by the combination of natural factors and human activities. LUCC is not only a manifestation of the process of balancing multiple driving factors but also the most intuitive manifestation of human activities changing the earth system [1]. Existing research shows that humans have changed about 30–50% of the global land surface, and the proportion has a high possibility to continue to increase with the needs of human survival and development [2]. With the continuous repetition and accumulation of LUCC in time and space, its impact on global climate change, biodiversity protection, ecosystem service supply, ecosystem structure and function, and the biogeochemical cycle has gradually appeared [3,4,5,6,7]. Therefore, dynamic monitoring research of LUCC is of great significance for discussing the sustainable development of human settlements and is a hot topic in the field of global change research.

LUCC can be divided into three types: modification (internal change of type), conversion (conversion between different types), and maintenance (maintaining a specific state) [8]. The type of change depends mainly on the combination of the various driving factors and the differences in spatial and temporal when the driving factors exert their effects [9]. Different combinations of driving factors, target objects, and time and space of action will produce different results of LUCC. Thus, sorting out the complicated relationship between the driving factors and LUCC and clarifying the impact magnitude and direction of the driving factors on LUCC are the core issues of LUCC research. On this basis, modeling and forecasting future LUCC scenarios will help guide decision-making [10,11].

LUCC is generally considered to be the main factor determining the landscape pattern on the surface and the most direct driving force for landscape pattern changes [12]. It affects the energy flow, material circulation, and ecological processes of regional ecosystems by changing the original landscape pattern, thereby participating in the global change process [13,14]. Calculating the landscape pattern index by describing land use/cover pattern characteristics has become an essential method for landscape spatial pattern analysis. Therefore, it is possible to observe the change of landscape pattern and its ecological process by analyzing the dynamic process of LUCC [15,16]. Increasing landscape spatial heterogeneity at specific scales, breaking the continuity of landscape patches, and aggravating the fragmentation of the overall landscape pattern are the main changing directions of landscape patterns caused by LUCC. This influence trend is more significant when human activities become the dominant factor driving LUCC [17,18]. Any subtle changes in land use may cause changes in the landscape pattern. However, different landscapes have different sensitivities to this change, so the effect of LUCC on landscape pattern changes will be different.

Cultivated land provides necessary material resources for human survival, life, and production. As the primary land use/land cover type and the vastest human-made landscape, it is an essential work of LUCC research to carry out dynamic monitoring of cultivated land pattern changes at different spatial and temporal scales [19,20]. Research shows that the area of global cultivated land has been increasing continuously in the past few centuries and showed a tendency to expand to higher latitudes [21,22]. The increase in the area of cultivated land can create higher grain output to ease the pressure of grain supply [23,24], but cultivation activities such as irrigation and fertilization will change the surface heat distribution and release greenhouse gases to affect local and global climate change [25,26]. The rapid growth of cultivated land is often carried out by encroaching on ecological land (forest land and grassland), which is the main driving force for reducing tropical rain forest areas worldwide [21,27,28].

On the contrary, cultivated land is a victim of increased construction land during the urbanization process in some areas [29,30]. This phenomenon is particularly prominent in some regions of China. Since the implementation of the reform and opening-up policy in China, the rapid expansion of construction land space along with the rapid economic development will inevitably encroach on the existing cultivated land, resulting in the continuous loss of limited cultivated land and seriously affecting the agricultural production potential [31]. Urban development occupied 2.26% of China’s cultivated land from 1990 to 2010 [32]. Meanwhile, China has acquired newly cultivated land to compensate for the decrease in cultivated land by land remediation and reclamation activities. The results show that the total area of cultivated land in China showed the changing characteristics of increasing in the north and decreasing in the south, and the center of gravity of the newly increased cultivated land moved to the northwest during this period [33]. Contrary to expectations, it not only changed the original cultivated land distribution pattern but also caused a decline in cultivated land quality [34,35]. This change in cultivated land pattern has reduced the total food energy production potential of China’s food crops by 9.64 × 10^12^ kcal in 20 years. Therefore, it is estimated that the output of food crops has decreased by about 34.9 million tons [36]. The enormous population base and the continuous growth trend have forced the issue of food security to become a vital issue that China needs to face for a long time. It is particularly important to understand the dynamic changes in the cultivated land pattern [37,38].

At present, researches on LUCC and landscape pattern changes mostly discuss the relationship between them and driving factors or ecological processes, and the interaction between them is relatively less. Analyzing and measuring the intensity and direction of LUCC’s impact on landscape pattern changes should become a bridge between LUCC research and landscape pattern change research. However, there are few research reports related to this problem in the existing research [18]. The objective of the present paper is to assess the characteristics of LUCC and quantify its impact on landscape pattern. We chose part of Dongting Lake Basin as the research area, which is a traditional farming area in China, and cultivated land is the most important type of land use. The study of LUCC in Dongting Lake area has been abundant. There are more researches on the pattern change of all land use types or the dynamic monitoring of the water pattern, but relatively fewer studies focus on the change of single cultivated land pattern. This paper analyzes the temporal and spatial changes and driving forces of cultivated land patterns in the Dongting Lake basin over a long time series in the nearly 40 years (1980–2018) since the implementation of China’s reform and opening-up policy. The primary purpose is to discuss the spatial and temporal change characteristics of cultivated land patterns and landscape patterns, the impact of driving factors on cultivated land changes, and the impact of cultivated land changes on landscape patterns. Our research is expected to provide a reference for the protection of regional cultivated land resources and contribute to the realization of regional sustainable development.

## 2. Materials and Methods

### 2.1. Study Area

**Figure 1:**
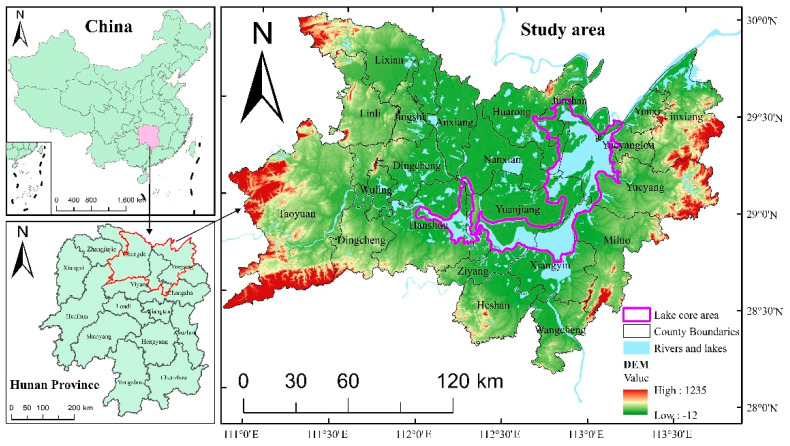
Location and topographic layout of the study area.

Dongting Lake is the second-largest freshwater lake in China, including the East Dongting Lake, the South Dongting Lake, and the West Dongting Lake. Its north is connected to the Changjiang River, and the south is connected to four main rivers, including Xiangjiang River, Zishui River, Yuanjiang River, and Lishui River, and many tributaries. The Dongting Lake Basin is part of the Yangtze River Basin, located in the middle reaches of the Yangtze River. The broad Dongting Lake Basin includes most parts of Hunan Province and parts of Hubei, Jiangxi, Guizhou, Chongqing, and Guangxi. The research areas (28° 08′–29° 58′ N, 110° 5′–113° 45′ E) selected in this paper are mainly located in the northern part of Hunan Province, including 21 county-level administrative regions, with a total area of about 3.12 × 104 km^2^ (Figure 1).

The research area, with Dongting Lake as the core, transitions to the east, west, and south directions in sequence to the river and lake alluvial plain, the hilly land around the lake, and the low-altitude mountain, forming a dish-shaped basin. Besides, this area has a subtropical monsoon climate, with a warm and humid climate, distinct seasons, and abundant rainfall. Its average annual precipitation is between 1200 and 1400 mm [39]. Because of its flat terrain and climatic conditions during the same period of rain and heat, the study area is extremely suitable for the cultivation of crops, especially rice, and has cultivated a large area of arable land [40].

### 2.2. Data Sources

**Figure 2:**
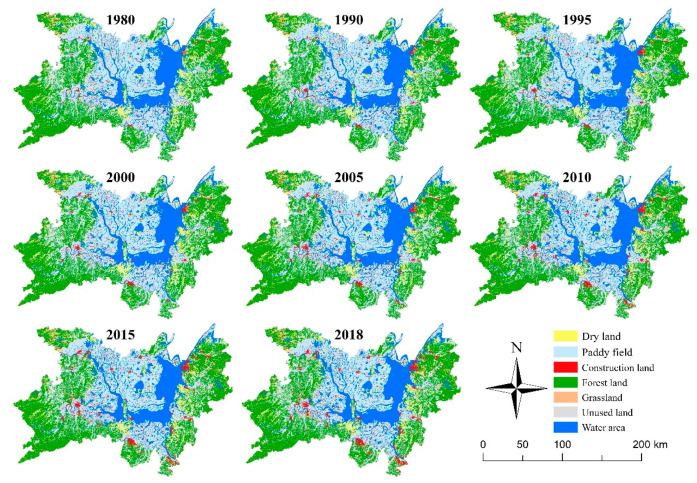
Landscape types of Dongting Lake Basin.

Remote sensing monitoring data (spatial resolution of 30m) of the land use status in 8 phases of 1980, 1990, 1995, 2000, 2005, 2010, 2015, and 2018 were selected as the underlying data of this study. The first 7 data come from the “Multi-period Land Use and Land Cover Remote Sensing Monitoring Data Set (CNLUCC)” provided by the Resource and Environmental Science Data Centre of the Chinese Academy of Sciences. With high credibility, they were collected on the resource and environment data cloud platform (http://www.resdc.cn/) [41]. The land use data in 2018 is based on the land use data in 2015, used Landsat 8 remote sensing image data as the primary information source, and generated by visual interpretation. The classification system of LUCC is mainly based on CNLUCC and refers to the standard Chinese classification of land use status (GB/T 21010-2017). We divide the land-use types of the study area into six first-class categories (Figure 2) and 19 s-class categories, such as cultivated land (paddy field, dry land), forest land (forest land, shrubbery, sparse forest land, and other forest land), grassland (high, medium and low coverage grassland), water area (river and canal, lake, reservoir pond, beach, and swampland), construction land (urban land, rural residential area and other construction land) and unused land (bare land and stone land).

Shuttle radar topography mission digital elevation model (SRTM DEM) data (90m) comes from the geospatial data cloud platform (http://www.gscloud.cn/), which is used to extract elevation data of the study area and generate regional slope data. In addition, the data of 14 national benchmarks and basic meteorological stations in and around the study area, from 1980 to 2018, were selected from the shared data provided by the National Meteorological Data Centre (http://www.nmic.cn/) Data set (V3.0). Finally, social and economic data such as population and gross domestic product (GDP) come from the statistical yearbook of Hunan Province in the corresponding year.

### 2.3. Methods

#### 2.3.1. Description Index of Cultivated Land Change

The traditional land use dynamic degree calculation may cause the loss of land change information because it only considers the changes in the total amount at the beginning and end of the period [42]. In order to avoid this problem, this paper integrates the annual average of the transferred-in amount (increased) and transferred-out amount (decrease) of cultivated land to reflect the active degree of the total change of cultivated land in the study period. The dynamic degree index of cultivated land change within a specific period is calculated as follows in Equation (1):

(1) $$DACLC_{i}=\frac{TO_{i}+TI_{i}}{TO_{i}+TI_{i}+S_{ii}}\times \frac{1}{T_{n}}\times 100\%$$

where *DACLC_i_* is the dynamic degree index that represents the change ratio of *i*-type cultivated land conversion. *TO_i_* denotes the area of land use changing from type *i* to other land types (including other cultivated land types). *TI_i_* denotes the area of land use changing from other land types (including other cultivated land types) to type *i*. *S_i_* is the area of *i*-type cultivated land that has not been converted, and *T_n_* is the number of years in the study period.

In order to better describe the distribution density of cultivated land, this paper constructs the distribution density index of cultivated land (*CLDDI*). It is calculated as follows in Equation (2):

(2) $$CLDDI_{ij}=\frac{BC_{ij}}{TC_{i}}\times \frac{TA}{BA_{j}}$$

where *CLDDI_ij_* is the cultivated land distribution index of the *j*-th buffer zone in phase *i*. *BC_i_* denotes the cultivated land area of the *j*-th buffer zone in phase *i*. *TC_i_* is the total area of cultivated land in all buffer zones, *TA* is the total area of buffer zones, and *BA_j_* is the area of the *j*-th buffer zone.

When *CLDDI* > 1, it indicates that the ratio of cultivated land area in the buffer zone is higher than the average cultivated land area in the study area. That is, the cultivated land distribution in the buffer zone is dense. However, when *CLDDI* < 1, it indicates that the cultivated land distribution in the buffer zone is relatively sparse. Besides, when *CLDDI* = 1, it indicates that the proportion of cultivated land area in the buffer zone is equal to the proportion of common cultivated land area in the study area.

#### 2.3.2. Trend Analysis

This paper uses the Sen trend degree and Mann-Kendall monotonicity test identification method based on the moving window to describe the spatial distribution characteristics of the trend (increase or decrease) of specific attribute values in more detail [43,44]. We used non-parametric linear regression Sen’s slope estimator, which can reduce noise interference, but it cannot realize the significance judgment of sequence trend. The Mann-Kendall method has no requirements for sequence distribution and is not sensitive to outliers, so the introduction of this method can complete the significance test of the sequence trend.

In this paper, this method is applied to identify the trend of cultivated land components and landscape pattern index. A square window with a grid of 17 × 17 (side length of 510 m) was selected as the basic unit, and the proportion of cultivated land area was calculated and assigned to the central grid. After that, it moves the 1-pixel unit at a time, starting from the upper left corner of the raster image, and forms a raster map of the moving window statistics. Finally, we get the raster image of the moving window statistics. With the help of geographic information system (GIS) tools, the grid results of 8 periods are superposed in space so as to extract eight attribute values of pixels in the same position and then form a sequence according to the time sequence of trend analysis.

The Mann-Kendall test statistic *S* is calculated as Equation (3):

(3) $$S=\left|\sum_{i=1}^{n-1}\sum_{j=i+1}^{n}\mathrm{sgn}\left(v_{j}-v_{i}\right)\right|$$

where *n* is the number of data points, *v_i_* and *v_j_* are the data values in time series *i* and *j* (*j > i*), respectively, and sgn(*x_j_ − x_i_*) is the sig function as follows in Equation (4):

(4) $$\mathrm{sgn}\left(v_{j}-v_{i}\right)=\{\begin{array}{ccc} 1 & \mathrm{if} & v_{j}-v_{i}>0\\ 0 & \mathrm{if} & v_{j}-v_{i}=0\\ -1 & \mathrm{if} & v_{j}-v_{i}<0 \end{array}$$

When *n* ≤ 10, the statistic *S* is used directly for the bilateral trend test, and we need to find the significance test probability *α* in the probability table according to *S* value [45]. If the probability value in the probability table corresponding to the computed *S* is less than the a priori specified *α* (in this paper *α* = 0.05) significance level of the test, we believe that there is a significant trend. After searching, we found that *α* is equal to 0.031 when *S* is equal to 16, and α is equal to 0.054 when *S* is equal to 14. If the *S* is not found in the probability table, the next value away from 0 in the table is used instead [45]. In summary, in this study, it means *α* ≤ 0.031 when *S* ≥ 15, which indicates that there is a significant monotonous change trend.

Sen’s slope estimator is computed as in Equation (5):

(5) $$\beta =Median\left(\frac{v_{j}-v_{i}}{j-i}\right)$$

where *β* is the changing trend of a pixel attribute value, *v_i_* and *v_j_* are the pixel values of the *i*-th and *j*-th phase of time series (*i* < *j*, *i*, *j* = 1, 2, …, 8), respectively. *β* > 0 indicates that the pixel attributes show an increasing trend in the study period, otherwise, *β* < 0 indicates a decreasing trend.

#### 2.3.3. Landscape Pattern Index

Landscape index refers to a simple quantitative index that can reflect some aspects of landscape structure composition and spatial configuration. The spatial arrangement and combination of landscape elements, as well as heterogeneity of landscape change characteristics, can also quantitatively express the relationship between landscape pattern and ecological process to a certain extent [46]. FRAGSTATS software version 4.2, (University of Massachusetts, Amherst, MA, U.S.) as a commonly used landscape pattern index calculation software, can reflect the basic features of landscape pattern in area-edge, shape, core area, contrast, aggregation degree, diversity, and other aspects from three levels of patch, class, and landscape [47]. We use the two methods, the static window method and the moving window method, to calculate the landscape pattern index. In particular, the mobile window function selects a 17 × 17 pixel (side length of 510 m) unit in a positive direction to calculate the landscape pattern index.

We conducted a preliminary screening of the landscape pattern index to avoid possible information redundancy between them [48]. For comparison, the four landscape indexes of area-weighted mean shape index (SHAPE_AM), mean fractal dimension index (FRAC_MN), patch density (PD), and patch cohesion index (COHESION) are selected at the class level and landscape level. They can reflect the basic characteristics of the regional landscape in terms of shape, fragmentation, and aggregation. The calculation method and ecological significance of these landscape indexes can be found in the relevant literature [47,49,50,51].

#### 2.3.4. Binary Logistic Regression

Binary logistic regression is a non-linear statistical method for regression modeling of binary-dependent and -independent variables (continuous or categorical variables), which is widely used in the analysis of driving mechanisms for regional LUCC and its forecasting [52,53]. In this study, the change of cultivated land is the dependent variable. During a research period, when the cultivated land is converted to other land types, its value is set to Y = 1; otherwise, Y = 0. Set the independent variables as *x*_1_, *x*_2_, *x*_3_, … *x*_n_, then the probability of cultivated land change is Equation (6):

(6) $$P\left(Y=1|x_{1},x_{2},x_{3}\cdots x_{n}\right)=\frac{\exp\left(\beta_{0}+\sum \beta_{n}x_{n}\right)}{1+\exp\left(\beta_{0}+\sum \beta_{n}x_{n}\right)}$$

The regression relationship is Equation (7):

(7) $$\log\mathrm{it}P\left(Y=1|x_{1},x_{2},x_{3}\cdots x_{n}\right)=\ln\left(\frac{P}{1-P}\right)=\beta_{0}+\beta_{1}x_{1}+\beta_{2}x_{2}+\cdots +\beta_{n}x_{n}$$

where *β*_0_, *β*_1_, …, *β_n_* are the regression coefficients. *P*/(1 − *P*) is called the odds of an event. Furthermore, the odds ratio (OR) of the two groups of events was obtained by division. When *β_n_* > 0 and statistically significant, it means that OR increases with the increase of the corresponding independent variable under the condition that other variables remain unchanged. On the contrary, when *β_n_* < 0 and statistically significant means that OR decreases as the corresponding independent variable increases. The goodness of fit of the regression model is tested by the receiver operating characteristic curve (ROC). When ROC ≥ 0.7, the situation indicates that the independent variable has strong confidence in the interpretation of the dependent variable [54].

## 3. Results

### 3.1. Cultivated Land Changes

#### 3.1.1. Changes in Cultivated Land Area

**Figure 3:**
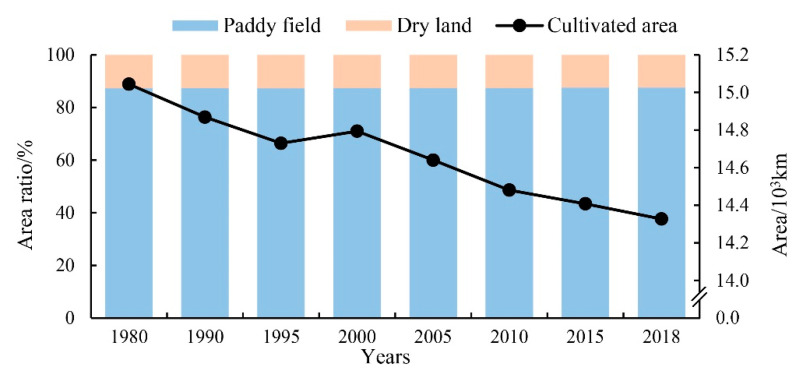
Change of cultivated land area in 1980–2018.

**Figure 4:**
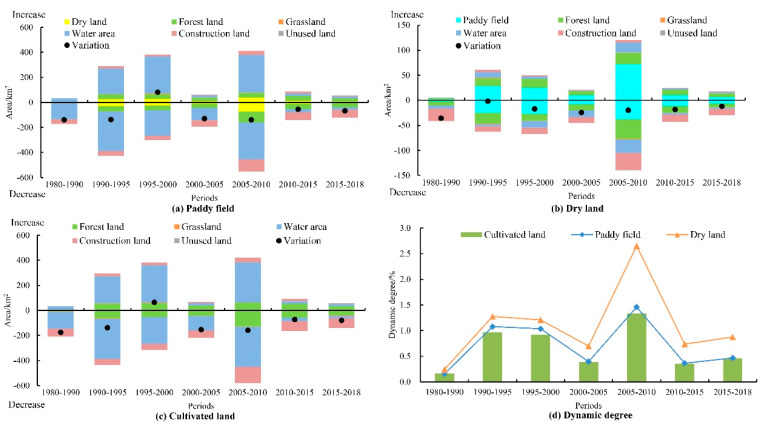
Dynamic degree of cultivated land change in 1980–2018. (**a**) conversion between paddy field and other land types; (**b**) conversion between dry land and other land types; (**c**) conversion between cultivated land and other land types; (**d**) dynamic degree of cultivated land change.

Cultivated land is the vastest land type in Dongting Lake Basin, accounting for 45.91% (in 2018) −48.21% (in 1980) of the total area of the study area. It can be divided into paddy field and dry land, and the area of paddy field is 6.83–7.01 times of that of dry land (Figure 3). The total area of cultivated land in the region showed a decreasing trend, from 15,043.73 km^2^ in 1980 to 14,327.60 km^2^ in 2018, a total decrease of 4.76% (or 716.13 km^2^), with an average annual decrease of 0.13%. The paddy field area has decreased from 13,124.87 km^2^ at the beginning of the period to 12,537.94 km^2^, a total of 4.47% (or 586.94 km^2^). Meanwhile, dry land has reduced from 1918.86 km^2^ to 1789.66 km^2^, a total of 6.73% (or 129.20 km^2^). Furthermore, dry land has decreased by 0.18% annually, higher than paddy field (0.12%). According to the changes in each period, paddy field and cultivated land have the same changing characteristics. Except for the increase in the total scale from 1995 to 2000, all other periods have decreased (Figure 4a,c). However, dry land showed a continuous decrease in the area throughout the study period (Figure 4b).

There was frequent and continuous conversion between cultivated land and other land use types, which made the regional land-use types in a dynamic changing process. During the study period, the active degree of total cultivated land area change was relatively stable, the dynamic degree of dry land was the highest, followed by paddy field, and the comprehensive dynamic degree of cultivated land was the lowest. Moreover, the overall trends of the dynamics of paddy field, dry land, and cultivated land were more consistent, showing the changing trend of the “M” shape (Figure 4d). Before 2010, the dynamics of paddy fields, dry land, and cultivated land generally showed an upward trend. They reached the maximum between 2005 and 2010 (1.46%, 2.65%, 1.33%), indicating that the conversion between cultivated land and other land types was active. After 2010, the dynamic degree of cultivated land declined rapidly and remained at a low level, indicating that the dynamic change of cultivated land tends to be stable.

The water area was the vastest land-use type with the mutual transformation of cultivated land, accounting for 60.69% (2072.73 km^2^) of the total area of cultivated land change (inward and outward). From 1980 to 2010, it was an active period of mutual transformation between cultivated land and water area, and the mutual transformation area decreased sharply after 2010. Among them, the transformation between paddy fields and water areas was particularly prominent, accounting for 94.49% (1958.46 km^2^) of the mutual transformation area between cultivated land and water areas. In particular, the mutual transformation between paddy field and water area was the primary form of realization of dynamic changes in paddy fields, accounting for 66.47% (1323.14 km^2^) and 56.49% (1910.07 km^2^) of the paddy fields transferred in and out.

The mutual transformation relationship between cultivated land and construction land was mainly manifested in the process of converting cultivated land into construction land, accounting for 24.50% (506.13 km^2^) of the total loss of cultivated land. The area of construction land converted into cultivated land accounted for 8.26% (111.48 km^2^) of the total increase of cultivated land, which is mainly converted from abandoned industrial and mining land to cultivated land through land consolidation project. This process has occurred mostly in the areas around towns and along the transportation lines, which was one of the main loss directions of the total cultivated land. Of the total losses, paddy field area accounted for 76.21%, and dry land area accounted for 23.79%.

The mutual transformation between cultivated land and forest land accounted for 22.76% (307.12 km^2^) and 19.29% (398.39 km^2^) of the total cultivated land transferred in and out, respectively. Destroying forest land and reclaiming it as cultivated land mainly occurred in the transition zone between plain, hill, and mountain. The conversion of cultivated land to forest land was mainly due to the large-scale cultivation of *Populus nigra* since the late 1990s, and it mainly took place in the west Dongting lake in Hanshou county and the surrounding area of south Dongting lake in Yuanjiang city. In addition, in order to control soil erosion, the project of returning cultivated land to forest land also promotes the conversion of cultivated land to forest land in some areas.

Because of their small area, the conversion between grassland and unused land and cultivated land accounted for only 0.53% and 0.04% of the total area of cultivated land conversion. In addition to the above, there was also frequent mutual conversion between paddy fields and dry land, which was the most important form of dry land change, accounting for 37.52% of the total area of dry land conversion. The overall performance was that the paddy field was converted into dry land (154.48 km^2^), which was slightly higher than that into paddy field (119.01 km^2^), reflecting a certain degree of degradation of cultivated land in the region.

#### 3.1.2. Spatial Distribution Characteristics of Cultivated Land

Dongting Lake is the core landscape of the study area and has an important impact on the ecological environment of the area. In order to analyze the impact of Dongting Lake on the distribution of cultivated land in the regional, the cultivated land was analyzed with a buffer zone centered on Dongting Lake. By extracting the water area in the eight periods and superimposing them spatially, we defined the core area of Dongting Lake (Figure 1) according to the principle of describing the maximum distribution range, coherence, and integrity of Dongting Lake. The area of the core area is 3021.11 km^2^. Then, the buffer zone is constructed by gradient with the core area as the center. Finally, a total of 26 buffer zones are divided from the inside out to demarcate the distribution area of cultivated land, and each of them radiates outward for 5 km.

**Figure 5:**
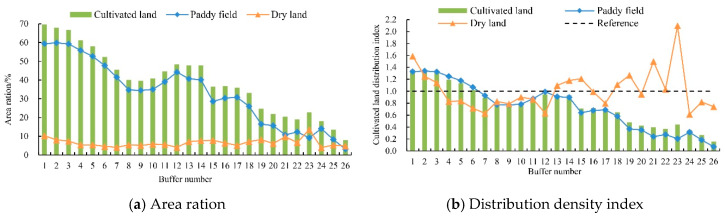
Distribution of cultivated land buffer zone. (**a**) proportion of cultivated land area to buffer area; (**b**) change of distribution density index.

According to the calculation, the standard deviation of the ratio of cultivated land to the total area of the buffer zone in each buffer zone in the eight periods was 0.020–1.660, and the standard deviation of the cultivated land distribution density index was 0.002–0.077. Because the indicator changed little throughout the study period, the average value of 8 periods was used to represent the overall trend. As shown in the figure (Figure 5a,b), the distribution pattern of cultivated land shows an obvious transition phenomenon in the process of radiating from the center of Dongting lake to the surroundings. Among them, the paddy field has a decreasing trend in both the number and density of distribution.

After a simple function fitting, the paddy field area ratio and the buffer zone number were in line with the linear function *y* = −2.188*x* + 62.207 (*R*^2^ = 0.923), and the distribution density index and the buffer zone number are in line with the linear function *y* = −0.049*x* + 1.392 (*R*^2^ = 0.923). Their fitting degree all performed well, and the monotony was significant. It showed that the farther away from Dongting lake, the smaller the distribution quantity and density of paddy fields were. Moreover, the distribution density of paddy fields was less than the average cultivated land density in the Dongting lake basin since the no. 7 buffer zone. On the other hand, the characteristics of such transitional changes in dry land were not prominent, and the fitting results of linear functions were *y* = 0.023*x* + 6.247 (*R*^2^ = 0.007) and *y* = 0.004*x* + 0.967 (*R*^2^ = 0.007). Their *R*^2^ values were too small, the goodness of fit was weak, and the trend of change was not significant. Besides, the overall variation characteristics of cultivated land were consistent with paddy fields because paddy field accounted for a high proportion of the total area of cultivated land.

#### 3.1.3. The Trend of Increase or Decrease

**Figure 6:**
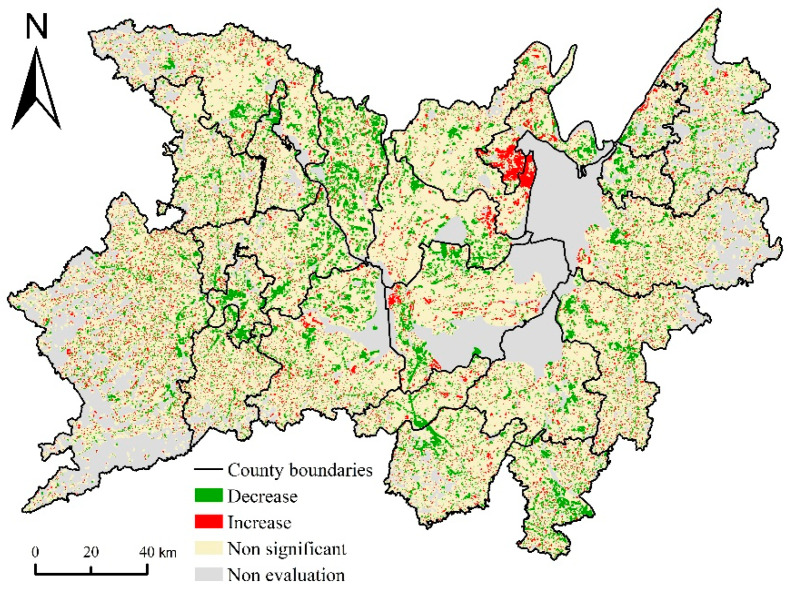
Trend analysis of increase and decrease

Excluding areas with no cultivated land pixel distribution in the 17 × 17 pixels window during the entire study period, a total of 78.32% (or 24,440.17 km^2^) of the study area became the evaluation area. The results showed (Figure 6) that the evaluation area of 78.75% (or 19,246.47 km^2^) did not pass the significance test of α < 0.05, indicating that the cultivated land area in this area had no evident trend of increase or decrease. Still, it was the main body of cultivated land distribution in the entire basin. In the evaluation area, 14.42% (or 3525.20 km^2^) of the area showed a significant decrease trend in cultivated land, while the significant increase area of cultivated land only accounted for 6.83%, reflecting the overall changing trend of decreasing cultivated land area in the basin.

The distribution areas of decreasing trend were mainly distributed around towns and along traffic lines in the plain region. For example, they were concentrated in Wuling, Anxiang, Yuanjiang, Yueyanglou, and Wangcheng. While, the distribution areas of increasing trend were mainly in areas close to water, where cultivated land can be reclaimed to take advantage of abundant water resources, especially in the surrounding area of Dongting lake. For example, they have formed obvious increasing agglomeration areas in Junshan and Huarong county on the west side of East Dongting Lake, which is an important manifestation of the shrinking area of Dongting Lake.

### 3.2. Driving Force Analysis of Cultivated Land Changes

#### 3.2.1. Driving Factors and Sampling

Considering the availability, computational feasibility, regional characteristics, and time continuity of the data, we selected spatial data of land use, natural environment data, and social and economic data to construct the index system of driving factors of cultivated land change in the research area. In order to avoid the impact of independent variable multicollinearity on the accuracy of the regression model, the continuous variables were tested by using SPSS software, and 17 driving factors were finally selected (Table 1). All the driving factors were processed and vectored in ArcGIS10.2 and re-sampled into raster data with a spatial resolution of 30 m.

We used the random point generation tool of ArcGIS for random sampling, and the distance between random points was set to be more than 100 m. First, random sample points with changes in cultivated land (*Y* = 1) in each period were extracted to obtain the maximum number of sampling points. Then, random sample points with invariable cultivated land (*Y* = 0) were extracted according to the same amount to avoid the negative impact of unequal proportional sampling on a coefficient estimation of explanatory variables in the Logistic regression model. The final sample points of each phase were 33,328, 148,688, 124,942, 126,916, 172,126, 142,722, and 95,680, respectively.

#### 3.2.2. Change of Driving Force

The results of logistic regression and ROC test (Table 2) showed that the percentage accuracy in classification was 65.95% to 89.25%, and the value of ROC was 0.723 to 0.945. These indicate that the regression model has a good fitting degree to the variable data and a good ability to explain the cultivated land change in Dongting Lake basin.

The factors of elevation (*x*_1_) and slope (*x*_2_) were negatively correlated with the turning out of cultivated land, indicating that the higher the altitude (or the greater the slope) was, the less likely the cultivated land would change. Also, the elevation of altitude has less influence on the occurrence ratio of cultivated land changes (*exp* (*β*) is close to 1). After 2010, *x*_1_ and *x*_2_ were no longer the main factors affecting the change of cultivated land, as they were eliminated from the regression model

The effect of climatic factors on the change of cultivated land was complex. In the study period, temperature (*x*_3_) showed an alternating change of “negative correlation–positive correlation” with cultivated land changes, and the effect was not significant after 2015. On the other hand, precipitation (*x*_4_) was negatively correlated with it. In general, the absolute values of the regression coefficients *β* of *x*_3_ and *x*_4_ gradually decreased, and the impact on the occurrence ratio of cultivated land changes gradually decreased. *x*_4_ had a small influence on the occurrence ratio of cultivated land changes. Each time *x*_4_ increased by 1 unit, *exp* (*β*) only changed by 2% × 4%. However, *x*_3_ had a more significant impact on the change of cultivated land. Each time *x*_3_ increased by 1 unit, *exp* (*β*) would decrease by 49.5% to 92.8% or increase by 1.63 to 9.16 times.

From the perspective of location conditions (*x*_5_–*x*_9_), the impact of each driving factor on the change of cultivated land had an obvious time difference. Before 2010, *x*_5_ and *x*_6_ were negatively correlated with changes in cultivated land. That is, the farther away from the core area of Dongting Lake and the water source, the less likely the cultivated land changes occurred. But they were opposite after 2010. Besides (*x*_7_), before 2005, the cultivated land far away from the urban area was more susceptible to change, mainly because of the project of returning farmland to forest and grass in the mountainous area. After 2005, it was shown that the cultivated land near the urban area was more likely to be occupied, which is an important manifestation of regional urbanization, and this trend is gradually strengthening. The driving factors *x*_8_ and *x*_9_ were roughly in the transition period from 2000 to 2010. There was a positive correlation with the transfer of cultivated land before 2000 but a negative correlation after 2010. This change process reflected the process of rural urbanization in the Dongting Lake Basin.

During the study period, the neighborhood enrichment (*x*_10_–*x*_13_) was positively correlated with the changes in cultivated land. That is, the higher the neighborhood enrichment was, the greater the possibility of the cultivated land changes would be. It indicates that cultivated land changes were more likely to occur at the marginal areas of cultivated land patches adjacent to other land use types than within the cultivated land patches. In particular, *x*_12_ and *x*_13_ had the strongest impact on cultivated land changes, mainly affected by policies such as returning cultivated land to forests or lakes and planting poplar (*Populus nigra*).

The impact of socio-economic factors (*x*_14_–*x*_17_) on the change of cultivated land was reflected in the driving effect of the total population (*x*_14_), per capita GDP (*x*_15_), and population growth rate (*x*_16_), which were in a positive proportion with cultivated land changes. *x*_14_ had little effect on it and remains unchanged (0.999–1.002 times), indicating that the regional population carrying capacity was good and could still accommodate the total size of the regional population. The impact of *x*_15_ on cultivated land changes showed a general downward trend with time, while the effect of *x*_16_ increased accordingly. The change rate of GDP (*x*_17_) had a significant impact on the change of cultivated land only before 2000, but no longer after 2000.

### 3.3. Landscape Pattern Changes

#### 3.3.1. Dynamic Changes in Landscape Pattern Index

**Figure 7:**
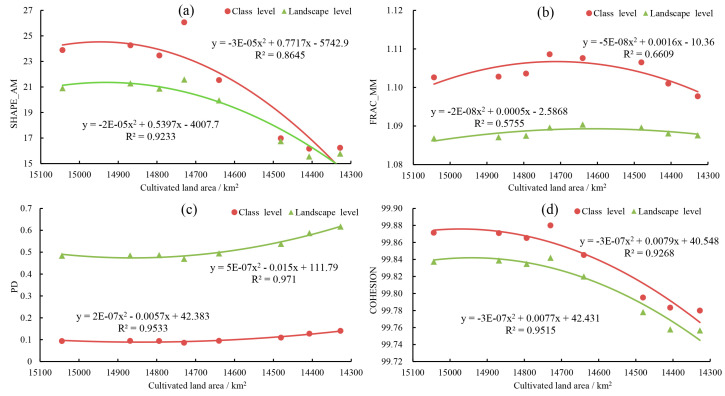
Relationship curve between landscape pattern index and cultivated land area by static window method. (**a**) SHAPE_AM, area-weighted mean shape index; (**b**) FRAC_MN, mean fractal dimension index; (**c**) PD, patch density; (**d**) COHESION, patch cohesion index.

The landscape pattern index calculated by the static window method can reflect the characteristics of the overall landscape pattern of the basin in Figure 7. The changing trend of landscape pattern index of cultivated land and the whole region was basically the same. SHAPE_AM and COHESION showed a trend of increasing first, then decreasing, and generally decreasing, with 1995 as the turning point at the class level and landscape level. It indicated that the physical connectivity of landscape decreases while the shape of cultivated land and overall landscape patch tended to be simple. The changing trend of PD was opposite to them, which was decrease first and then increase, and overall rise. However, the changing trend of PD was opposite to them, showing that the degree of fragmentation of cultivated land and the overall landscape first decrease and then increase. FRAC_MN of cultivated land was higher than that of the overall landscape. Obviously, the impact of human activities on the patch shape change of cultivated land was less than the average level of the basin. In particular, FRAC_MN continued to decrease after 2005, indicating that human activities continued to strengthen the transformation of the regional landscape.

**Figure 8:**
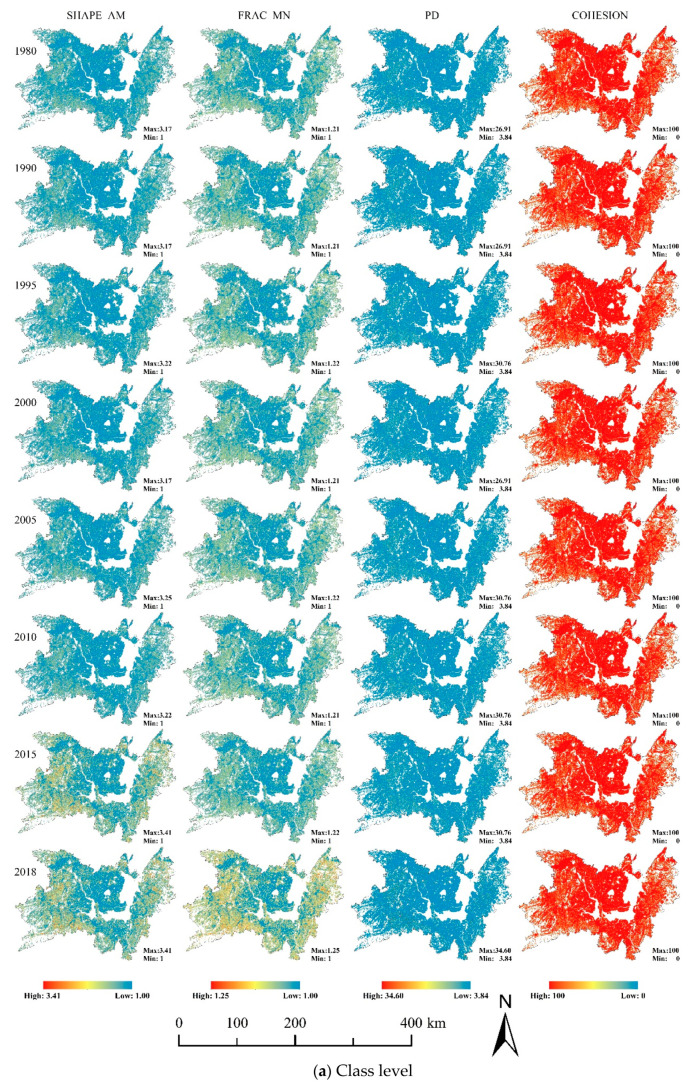

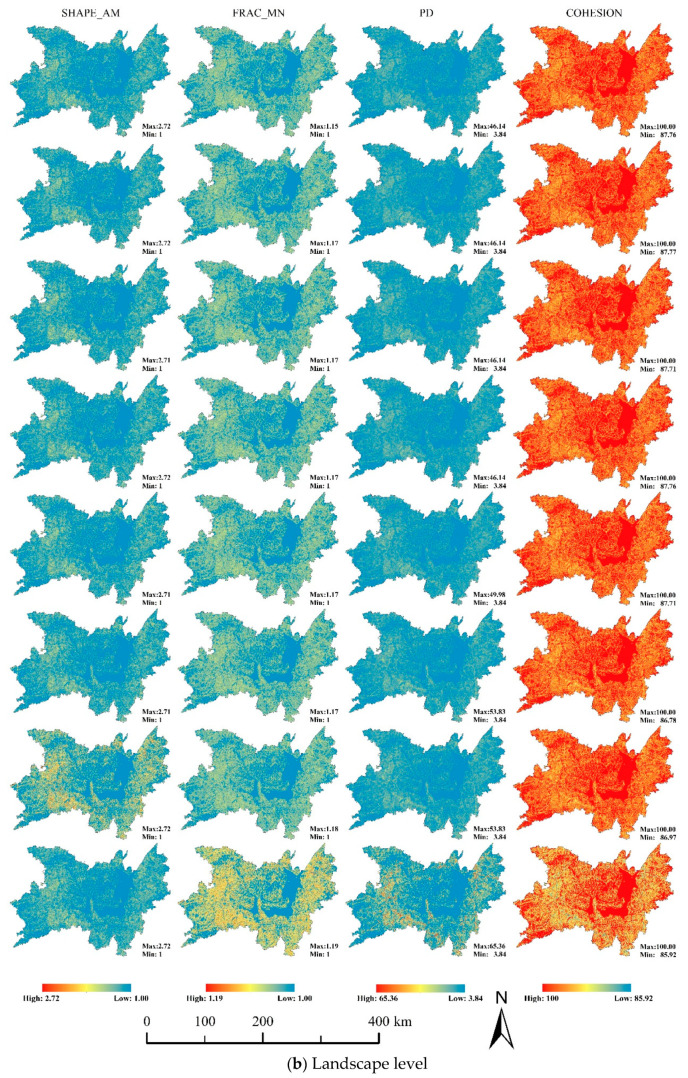
Change of landscape pattern indexes by moving window method. (**a**) Class-level landscape pattern indexes change; (**b**) Landscape-level landscape pattern indexes change.

The dynamic window method was used to reflect the local distribution characteristics of the landscape pattern index. Overall, the index values of SHAPE_AM, FRAC_MN, and PD were concentrated in the low-value areas of each index value range, while the COHESION was mainly concentrated in high-value areas in Figure 8. In terms of spatial distribution, the cultivated land and the overall regional landscape pattern index showed the characteristics of a gradual transition from Dongting Lake to surrounding areas. The surrounding area of Dongting Lake was a low-lying plain with a large area of contiguous cultivated land, so the landscape patches were in regular shape, with a low degree of fragmentation and good connectivity. However, the periphery of the study area was a mountainous area with rugged terrain and a significant slope. Therefore, the landscape patches here were complex in shape, relatively fragmented, and poor in connectivity.

**Figure 9:**
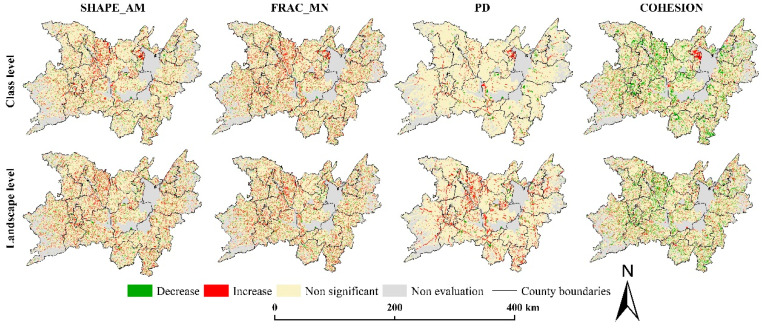
Trend analysis of landscape pattern index (SHAPE_AM, area-weighted mean shape index; FRAC_MN, mean fractal dimension index; PD, patch density; COHESION, patch cohesion index).

The trend analysis method could further reflect the increase or decrease of the landscape pattern index in local areas (Figure 9). The evaluation area accounted for 77.37% of the total area of the study area (or 24,144.55 km^2^). Most areas of the study area have no significant changes (α ≥ 0.05) in the landscape pattern index. At the level of cultivated land landscape, the areas with no significant increase or decrease in SHAPE_AM, FRAC_MN, PD, and COHESION accounted for 94.20%, 84.81%, 80.70%, and 78.59% of the evaluation area. Meanwhile, the proportions of these were 88.30%, 80.58%, 80.25%, and 78.79% at the landscape level. Furthermore, the areas of SHAPE_AM, FRAC_MN, and PD all showed the increasing trend was higher than the decreasing trend at the level of cultivated land and landscape, which accounted for 3.64%, 9.09%, 11.81%, and 9.03%, 11.64%, 11.57%. On the contrary, COHESION showed that the decreasing trend area is larger than the increasing trend area, and the decreasing trend area accounts for 14.27% and 13.82% at the level of cultivated land and landscape. We found that the significant change of landscape pattern index was mainly distributed in three areas: Huarong and Junshan junction on the west side of east Dongting lake, Anxiang, Dingcheng, and Hanshou on the north of west Dongting lake, and Wangcheng district. Besides, strip-like areas with significant change were formed along the traffic lines.

#### 3.3.2. Correlation Analysis

Based on the results of landscape pattern index of the moving window, Matlab was used to calculate the correlation and significance of landscape pattern index between cultivated land and the overall landscape (Table 3). The results showed that there was a significant positive correlation between them (*p* < 0.01). Their SHAPE_AM and FRAC_MN had a strong correlation, with a correlation coefficient of about 0.8, but it decreases gradually with time. The positive correlation of their PD gradually increased, and the correlation coefficient increased from 0.603 to 0.621, both. In terms of COHESION, there is only a very weak correlation.

**Figure 10:**
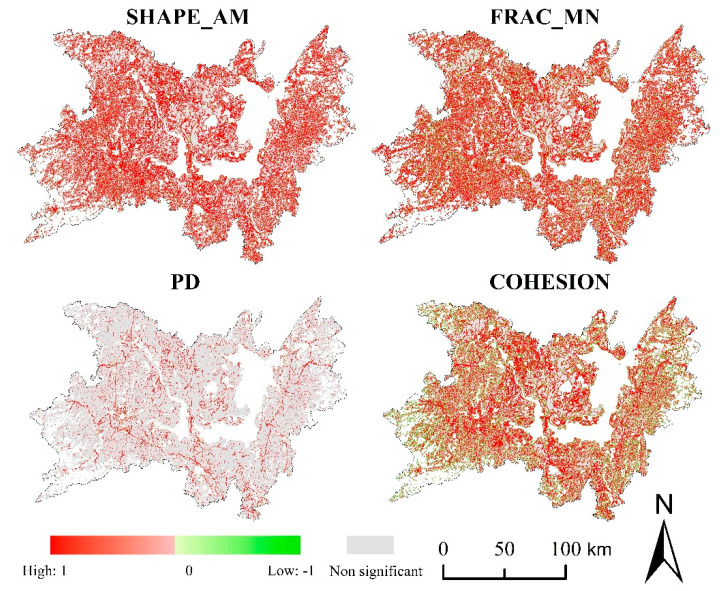
Time-series correlation of landscape pattern index (SHAPE_AM, area-weighted mean shape index; FRAC_MN, mean fractal dimension index; PD, patch density; COHESION, patch cohesion index).

Based on the time-series changes of the landscape pattern index on the same pixel in 8 periods, MATLAB was used to calculate the time series correlation and significance of the cultivated land and the overall landscape to reflect the correlation between the two levels at different spatial positions (Figure 10). We determined a total of 25,508,021 evaluation pixels, accounting for 73.57% (or 22,957.22 km^2^) of the total area of the study area after ensuring that the landscape index is non-null in the entire time series. Among the results of significance analysis, *p* < 0.05 was regarded as a significant correlation, and the rest were uncorrelated.

In the three indexes of SHAPE_AM, FRAC_MN, and COHESION, the area of significant correlation accounted for 52.87%, 61.67%, and 58.05% of the total area of the evaluation area, while PD was only 11.04%. Furthermore, each landscape pattern index is dominated by a positive correlation in the significant correlation, and the proportion of positive correlation areas reaches 93.60%, 86.27%, 99.02%, and 72.86%, respectively. Besides, FRAC_MN and COHESION have a relatively high area ratio of negatively significant correlation areas. The negatively significant correlation area of FRAC_MN was mainly distributed near construction land and located in the area of decreasing trend of cultivated land (Figure 7). The disappearance of cultivated land converted into construction land caused the decrease of FRAC_MN of the cultivated land patches in the local area, while the increase of the construction area caused the increase of FRAC_MN of the local patches at landscape level, which damaged the integrity of the landscape. The negatively significant correlation area of FRAC_MN was mainly distributed in mountainous and hilly areas and also located in the area of decreasing trend of cultivated land. Cultivated land in this area was mainly converted to forest land, so that the forest land patches could be expanded to the periphery and connected with other forest land patches, increasing the integrity of the regional landscape.

#### 3.3.3. Landscape Pattern Changes Caused by Cultivated Land Changes

The results of cultivated land changes (Figure 6) and landscape pattern index changes (Figure 9) were spatially superimposed, and the area ratio was counted to analyze the impact of cultivated land changes on regional landscape changes. It can be seen from Table 4 that in areas where the cultivated land change is not significant, the landscape pattern index changes are also not visible. There is no significant change trend in the cultivated land landscape pattern index in more than 90% of the area, and there is no significant change trend in the overall landscape pattern index of about 90% of the area. The proportion of the increased area and the decrease area of the cultivated land landscape pattern index were about the same. The area ratio of the increased area of SHAPE_AM, FRAC_MN, and PD was slightly larger, while COHESION was slightly smaller. However, at the landscape level, the difference between the increased area and the decrease area is vast, which is caused by the change of other landscape types except for cultivated land.

In the distribution areas of decreasing trend of cultivated land, the area ratio of SHAPE_AM, FRAC_MN, and PD increased area reached 28.46%, 49.23%, and 12.82%, respectively, which was significantly higher than that of the reduced area at class level of cultivated land. The area ratio of the COHESION decrease area reached 88.51%, indicating that the decrease of the cultivated land area would reduce the connectivity of the cultivated land landscape. At the landscape level, the changing trend of each index was the same as the cultivated land level. However, the area ratio of the COHESION decrease area was not as prominent as the cultivated land level, only 42.87%. It showed that in terms of landscape connectivity, other types of landscape pattern changes weakened the impact of cultivated land changes on regional landscape pattern changes.

In the distribution areas of the increasing trend of cultivated land, the changes of SHAPE_AM, FRAC_MN, and COHESION were opposite to the cultivated land decrease area but the same changes of PD at the cultivated land level. The changing trend of each landscape index was opposite to that of the cultivated land decrease area. The difference in area ratio between each index increase area and decrease area was small, and there was no evident tendency to increase or decrease at the landscape level.

## 4. Discussion

### 4.1. Complex Driving Force of Cultivated Land Change

There are apparent differences in the effectiveness of different driving factors, and the effectiveness of the same driving factor will also change with time and space [55,56]. Taking the Dongting Lake basin as an example, the warm and humid climatic conditions and distinct topographical features (Figure 1) lay the basic cultivated land structure based on paddy field (Figure 3) and the overall distribution pattern gradual transition from Dongting Lake as the center to the surroundings the overall distribution pattern (Figure 5). Since the implementation of the reform and opening-up policy, the influence of natural factors on the change of cultivated land pattern has gradually decreased, while the influence of human activities has gradually increased (Table 2). The rapid economic development has promoted the conversion of cultivated land with low economic benefits into construction land (or economic forests, breeding pond) with high economic output. At the same time, the continued growth of the population has led to increasing demands for residential land. The expansion of urban and rural settlements has eroded the surrounding cultivated land, and the construction of roads, railways, and other infrastructure facilities also occupied a large amount of cultivated land.

In addition to the demographic and economic factors selected for easier vectorization, many factors such as engineering, technology, and policy will also have an important impact on cultivated land changes. However, these driving forces are often difficult to express in vector, and most of them are discussed from the perspective of qualitative analysis [57,58]. For a long time, reclaiming land from the lakes has continuously transformed the lakes into paddy fields, which is the leading way to supplement the cultivated land in the region. On the other hand, the construction of farmland water conservancy facilities and large-scale freshwater aquaculture have promoted the conversion of paddy fields to river channels and reservoir ponds. Since the floods of the Yangtze River in 1998, the government has promoted the project of Converting Farmland to Lake (CFTL) to restore cultivated land formed by landfill lakes to lakes or plant poplar forests in Dongting Lake Basin [59]. On the one hand, CFTL slowed down the process of transforming waters into paddy fields; in particular, it significantly inhibited the increase of cultivated areas in the area around Dongting Lake. On the other hand, it also changed the water area that was mainly dominated by lakes into a paddy field and turned it into the water area dominated by reservoirs, ponds, and beaches. In order to pursue economic benefits, in the late 1990s, poplar planting activities were carried out in the Dongting lake basin, which was one of the main ways to transform cultivated land into forest land during this period [60]. In addition, the implementation of policies such as the balance of cultivated land occupation and reclaiming of abandoned industrial and mining land are an essential guarantee for the supplement of cultivated land area.

The change process of cultivated land in the Dongting Lake Basin showed that stakeholders’ preferences and willingness to pay played an important role. The stakeholders often change the original preferences and willingness to pay after weighing the relationship between rights and benefits, thereby leading the direction of land use change to a certain extent [61,62]. This trade-off between rights and interests usually takes economic benefits as a prerequisite, making the pursuit of land use with higher economic output a preference for landowners [63]. The government needs to consider the fundamental rights of the original beneficiaries of the land and the willingness to pay for changing the land use types when leading regional land use changes. Therefore, economic compensation has become the basic guarantee for guiding changes in land use types and successfully completing the goals of land use planning. Especially when it comes to scope conflicts between agricultural production areas and ecological reserves, how to achieve the balance of rights and obligations between nature reserve managers and resource users is a key issue that needs to be fully considered.

The comprehensiveness and complexity of land use changes indicate that it is difficult to calculate the contribution of each driving factor to the change of a single land type pattern independently. Try to get rid of the interference of other driving factors to purely establish the quantitative relationship between the change of a single driving factor and the change of a single land type, which helps to analyze the internal mechanism of land cover change under the condition of multi-factor driving. In a modern society where human factors gradually dominate, rationalizing vectorization of socio-economic indicators and reducing the spatial expression scale as much as possible will help to accurately evaluate the degree of impact of human activities on surface changes. It provides a scientific basis for better management and protection of regional ecological balance and the realization of regional sustainable development.

### 4.2. Impact of Cultivated Land Changes on Landscape Pattern

LUCC directly drives changes in landscape patterns, thereby affecting the structure and function of regional ecosystems [64]. From the overall analysis of the study area, under the general trend of continuous decrease in cultivated land area, the SHAPE_AM, FRAC_MN, and COHESION showed an overall decreasing change while PD increasing at the cultivated land level and landscape level (combined Figure 3 and Figure 7). However, the changing characteristics of the landscape pattern index of the moving window method are not identical. We believe that it is the result of the scale effect in the process of landscape pattern analysis [65,66]. From the perspective of the overall change, the change characteristics of the cultivated land landscape pattern index and the overall landscape pattern index are basically the same, showing a significant positive correlation. That is, the change of the cultivated land landscape pattern has a positive impact on the overall regional landscape pattern change (Figure 7, Table 3). From the perspective of local changes, there is a significant correlation between the cultivated land landscape pattern index and the overall landscape pattern index, and it is mainly positively correlated (Figure 10). This result shows that land use changes can not only directly drive the change of the overall landscape pattern of the region, but also indirectly trigger the change of the overall landscape pattern by driving a single type of landscape pattern change.

1995 was a significant turning point in the change of regional landscape pattern showing opposite trends in the two periods. Before 1995, the reduction of the cultivated land area increased the complexity of patches, weakened the degree of landscape fragmentation, and changed the landscape pattern with enhanced connectivity. It was the opposite after 1995. Under the same conditions of continuous decrease of cultivated land, this opposite change characteristic is caused by the difference in location characteristics of the cultivated land reduction area. It is mainly the outer boundary areas and the sporadic cultivated land that is transformed into other land types before 1995. However, after 1995, the internal area of continuous farmland and large-scale farmland patches also began to change to other land types.

This study believes that not only changes in the area of land use types will cause changes in the landscape pattern index, but changes in their spatial location will also cause changes in the landscape pattern index. Different from using the regression model to quantitatively describe the relationship between the area change of land use type and the change of landscape pattern [18], we take the cultivated land type as an example and try to use the area ratio to describe the probability of the directional change of landscape pattern index caused by the change of cultivated land through superposing the change trend result of cultivated land and the result of landscape index.

It can be seen from Table 4 that the reduction of local cultivated land area has a higher probability of increasing the complexity of the shape of the cultivated land patch, deepening the degree of fragmentation of the cultivated land landscape and weakening the landscape connectivity, which will also cause the same change in the overall landscape pattern of the region (Table 4). This is because, in the process of reducing the cultivated land area, it is not uncommon for large areas of continuous cultivated land to be converted to other land-use types. Instead, it is mainly dominated by the decrease and change of small area and sporadic cultivated land. Moreover, the spatial position of the reduced cultivated land area is more scattered, thereby destroying the integrity of the regional landscape. The increase of cultivated land area is more likely to cause the increase of FRAC_MN, PD, and COHESION, and the effect on SHAPE_AM is not significant. The increase of cultivated land area comes from the conversion of other land-use types adjacent to existing cultivated land. This transformation is a directional transformation under the strong influence of human activities so that the shape of the patch of cultivated land is more regular, and the physical connectivity is improved. However, there is a small difference between the proportion of increasing area and decreasing area in the index of overall landscape pattern in the cultivated land increasing area. This is mainly because the increased area of cultivated land is small, so the influence on the overall landscape pattern is small.

## 5. Conclusions

By analyzing the cultivated land changes, landscape pattern changes and driving force change in Dongting lake basin with a long time series, this paper aims to explain the regional change cycle process in which driving factors drive LUCC, LUCC causes landscape pattern change, and landscape pattern change affects the effect of driving factors. The results showed that from 1980 to 2018, the total cultivated land area in Dongting Lake Basin showed a trend of continuous decrease, which was mainly converted into construction land and water area. This change led to an increase in the degree of fragmentation of the regional landscape, an increase in the complexity of the shape of the landscape patches, and a decrease in the physical connectivity between them. The result of the landscape pattern change caused by LUCC is not only related to the area change of land use type but also closely related to the spatial location of the change. The driving force of cultivated land change changes with time, and human activities gradually replace natural factors to become the leading factor affecting the cultivated land change in the study area. The rapid development of the economy and the continuous growth of the population strongly and rapidly change the current situation of the surface, break the original landscape pattern, and cause a series of environmental and ecological changes, which put forward higher requirements for the realization of sustainable development. Understanding the preferences of different groups of people and measuring people’s willingness to pay in different situations will help improve the existing compensation mechanism, scientifically formulate compensation standards, and realize the sustainable development of social economy and environment.

## Figures and Tables

**Table 1:** Driving factors of cultivated land changes

Driving Factors	Unit	Describe
Elevation (*x*_1_)	m	Directly obtained DEM data.
Slope (*x*_2)_	1–5	The slope was calculated from DEM data. The slope was classified into 2°, 6°, 15°, and 25° as the discontinuous points (including upper but not lower) and assigned values of 1, 2, 3, 4, and 5.
Temperature (*x*_3_)	°C	We interpolated the annual average temperature (or annual precipitation) of each meteorological station by kriging to obtain the grid data of annual average temperature (or annual precipitation) with a spatial resolution of 30 m, and finally calculated the grid data of continuous average temperature (or annual precipitation) for many years during the research period.
Precipitation (*x*_4_)	mm	
Distance to Dongting Lake (*x*_5_)	km	Calculate the nearest direct distance of each grid to Dongting lake (the core area in Figure 1)
Distance to the water source (*x*_6_)	km	The rivers and canals, lakes, and reservoirs on the maps of land use types in different periods (Figure 2) were extracted as irrigation water sources. Then calculate the direct distance from each grid to the water source.
Distance to town (*x*_7_)	km	Extract the urban land on the land use type map of each period (Figure 2) and calculate the nearest distance of each grid to it.
Distance to a rural residential area (*x*_8_)	km	Extract the rural residential area on the land use type map of each period (Figure 2) and calculate the nearest distance of each grid to it.
Distance to other construction land (*x*_9_)	km	Extract the other construction land area on the land use type map of each period (Figure 2), mainly transportation land, mining area, industrial zone, etc., and calculate the nearest distance of each grid to it.
Neighborhood enrichment of grassland (*x*_10_)	/	The first-class land use type was extracted from the land use map of each period, and the neighborhood abundance index was calculated according to the radius of 5 × 5 pixels. The cultivated land neighborhood abundance was not included in the index system because of its collinear with other driving factors. Similarly, unused land was not included in the indicator system due to its small distribution range and area.
Neighborhood enrichment of construction land (*x*_11_)	/	
Neighborhood enrichment of forest land (*x*_12_)	/	
Neighborhood enrichment of water area (*x*_13_)	/	
Population density (*x*_14_)	cap/km^2^	The total resident population of each county divided by the county area.
GDP per capita (*x*_15_)	10^3^yuan/cap	The total GDP of each county divided by the total resident population of the county.
Rate of population change (*x*_16_)	%	It was calculated from the total population data of each period.
Rate of GDP change (*x*_17_)	%	It was calculated from the total GDP data of each period.

**Table 2:** Logistic regression results of cultivated land change in seven time series.

Driving Factors	1980–1990		1990–1995		1995–2000		2000–2005		2005–2010		2010–2015		2015–2018	
	*β*	exp(*β*)	*β*	exp(*β*)	*β*	exp(*β*)	*β*	exp(*β*)	*β*	exp(*β*)	*β*	exp(*β*)	*β*	exp(*β*)
*x*_1_	−0.006	0.994	−0.004	0.996	−0.003	0.997	*	*	−0.003	0.997	*	*	*	*
*x*_2–1_	−4.078	0.017	−2.668	0.069	−1.399	0.247	−0.610	0.543	−2.018	0.133	*	*	*	*
*x*_2–2_	−4.740	0.009	−3.066	0.047	−1.943	0.143	−0.867	0.420	−2.295	0.101	*	*	*	*
*x*_2–3_	−4.089	0.017	−2.797	0.061	−1.553	0.212	−0.929	0.395	−2.096	0.123	*	*	*	*
*x*_2–4_	−2.087	0.124	−2.051	0.129	−0.849	0.428	−0.721	0.486	−1.362	0.256	*	*	*	*
*x*_3_	−2.628	0.072	2.215	9.162	−2.029	0.131	0.489	1.631	−0.684	0.505	0.659	1.932	*	*
*x*_4_	0.004	1.004	−0.003	0.997	−0.002	0.998	−0.001	0.999	−0.002	0.998	−0.002	0.998	0.001	1.001
*x*_5_	−0.024	0.976	−0.014	0.986	−0.006	0.994	−0.003	0.997	−0.005	0.995	0.010	1.011	0.002	1.002
*x*_6_	−0.177	0.838	−0.056	0.945	−0.053	0.948	−0.020	0.980	−0.056	0.946	0.045	1.046	0.034	1.034
*x*_7_	−0.013	0.987	*	*	0.010	1.010	0.008	1.008	−0.015	0.985	−0.037	0.964	−0.043	0.958
*x*_8_	0.161	1.174	0.439	1.551	0.020	1.020	−0.075	0.927	0.102	1.108	−0.015	0.985	*	*
*x*_9_	−0.008	0.992	0.026	1.026	0.014	1.014	−0.006	0.994	*	*	−0.034	0.966	−0.039	0.962
*x*_10_	0.055	1.057	0.118	1.125	0.088	1.092	0.104	1.109	0.072	1.074	0.096	1.101	0.096	1.100
*x*_11_	0.065	1.067	0.259	1.296	0.167	1.182	0.256	1.292	0.183	1.200	0.368	1.445	0.358	1.430
*x*_12_	0.530	1.698	3.028	20.648	2.691	14.751	2.972	19.531	2.037	7.670	3.289	26.810	3.023	20.551
*x*_13_	0.720	2.055	1.351	3.863	1.283	3.608	1.615	5.028	1.158	3.184	1.964	7.128	1.947	7.006
*x*_14_	0.001	1.001	−0.001	0.999	0.002	1.002	0.000	1.000	0.001	1.001	0.000	1.000	0.001	1.001
*x*_15_	0.195	1.215	0.128	1.136	−0.078	0.925	0.036	1.037	0.015	1.015	0.020	1.021	0.002	1.002
*x*_16_	−0.003	0.997	0.005	1.005	0.010	1.010	−0.021	0.979	−0.017	0.983	−0.023	0.978	0.041	1.042
*x*_17_	0.003	1.003	−0.002	0.998	−0.001	0.999	*	*	*	*	*	*	*	*
ROC	0.723		0.882		0.866		0.889		0.803		0.945		0.922	
Percentage correct 0	70.96		83.50		86.67		85.32		83.84		87.88		85.70	
Percentage correct 1	60.94		78.55		74.30		83.52		65.36		90.62		86.99	
Overall percentage	65.95		81.02		80.49		84.42		74.60		89.25		86.35	

Notes: * means that the independent variable statistical test is not significant. Others pass the 95% confidence significance test.

**Table 3:** Correlation of landscape pattern index between class and landscape level.

Landscape Pattern Index	1980	1990	1995	2000	2005	2010	2015	2018
SHAPE_AM	0.812	0.809	0.814	0.808	0.808	0.803	0.798	0.800
FRAC_MN	0.816	0.813	0.817	0.812	0.810	0.804	0.801	0.802
PD	0.603	0.602	0.603	0.600	0.605	0.613	0.615	0.621
COHESION	0.062	0.056	0.057	0.055	0.054	0.059	0.063	0.067

Notes: SHAPE_AM, area-weighted mean shape index; FRAC_MN, mean fractal dimension index; PD, patch density; COHESION, patch cohesion index.

**Table 4:** Correlation of landscape pattern index between class and landscape level (%).

Trend of Cultivated Land	Trend of Landscape Pattern Index	Class Level				Landscape Level			
		SHAPE_AM	FRAC_MN	PD	COHESION	SHAPE_AM	FRAC_MN	PD	COHESION
Non-significant 78.71%	Non-significant	91.36	91.38	97.62	96.71	88.24	87.47	93.78	88.04
	Decrease	3.64	3.69	0.82	1.81	4.80	5.27	1.43	7.60
	Increase	5.00	4.92	1.56	1.48	6.96	7.26	4.79	4.36
Decrease 14.45%	Non-significant	57.11	39.72	77.63	10.73	51.1	52.72	60.20	42.27
	Decrease	14.42	11.05	9.55	88.51	16.64	17.14	6.67	42.87
	Increase	28.46	49.23	12.82	0.76	32.25	30.14	33.12	14.86
Increase 6.83%	Non-significant	67.96	44.36	89.87	13.39	54.76	55.36	84.56	49.47
	Decrease	16.84	43.73	1.94	0.81	23.32	22.76	8.49	24.04
	Increase	15.21	11.91	8.19	85.8	21.92	21.87	6.95	26.49

Notes: SHAPE_AM, area-weighted mean shape index; FRAC_MN, mean fractal dimension index; PD, patch density; COHESION, patch cohesion index.
